# TXNL6 Is a Novel Oxidative Stress-Induced Reducing System for Methionine Sulfoxide Reductase A Repair of α-Crystallin and Cytochrome C in the Eye Lens

**DOI:** 10.1371/journal.pone.0015421

**Published:** 2010-11-04

**Authors:** Lisa A. Brennan, Wanda Lee, Marc Kantorow

**Affiliations:** Biomedical Sciences Department, Charles E. Schmidt College of Medicine, Florida Atlantic University, Boca Raton, Florida, United States of America; University of Florida, United States of America

## Abstract

A key feature of many age-related diseases is the oxidative stress-induced accumulation of protein methionine sulfoxide (PMSO) which causes lost protein function and cell death. Proteins whose functions are lost upon PMSO formation can be repaired by the enzyme methionine sulfoxide reductase A (MsrA) which is a key regulator of longevity. One disease intimately associated with PMSO formation and loss of MsrA activity is age-related human cataract. PMSO levels increase in the eye lens upon aging and in age-related human cataract as much as 70% of total lens protein is converted to PMSO. MsrA is required for lens cell maintenance, defense against oxidative stress damage, mitochondrial function and prevention of lens cataract formation. Essential for MsrA action in the lens and other tissues is the availability of a reducing system sufficient to catalytically regenerate active MsrA. To date, the lens reducing system(s) required for MsrA activity has not been defined. Here, we provide evidence that a novel thioredoxin-like protein called thioredoxin-like 6 (TXNL6) can serve as a reducing system for MsrA repair of the essential lens chaperone α-crystallin/sHSP and mitochondrial cytochrome c. We also show that TXNL6 is induced at high levels in human lens epithelial cells exposed to H_2_O_2_-induced oxidative stress. Collectively, these data suggest a critical role for TXNL6 in MsrA repair of essential lens proteins under oxidative stress conditions and that TXNL6 is important for MsrA defense protection against cataract. They also suggest that MsrA uses multiple reducing systems for its repair activity that may augment its function under different cellular conditions.

## Introduction

Significant evidence points to a major role for protein oxidations in the etiology of many age-related human degenerative disorders including Alzheimer's disease [1]–[2], Parkinson's disease [3]–[5], and age-related cataract of the eye lens [6]. Protein oxidation can result in altered conformation, activity, sub-cellular localization patterns, and aggregation states which are associated with loss of cellular functions, apoptosis, and cell death [7]. Proteins become oxidized upon exposure to reactive oxygen species (ROS). Exogenous sources of ROS include environmental oxidants, radiation and drugs [8]. Endogenous ROS can arise as a by-product of mitochondrial respiration through inefficient electron coupling at complexes I and III of the electron transport chain [9]–[10]. ROS levels increase upon aging as a consequence of multiple events including age-related accumulation of mitochondrial mutations, resulting from exposure to endogenous ROS [11]. The two most common protein oxidations upon aging and disease are oxidation of cysteines and methionines [7] [12] [13].

Protein methionines (mets) are rapidly oxidized to form protein methionine sulfoxides (PMSO) upon exposure to hydrogen peroxide, hydroxyl radical, and other sources of ROS [13]. In the eye lens, PMSO levels increase upon aging [14] and in human cataractous lenses 60%–70% of total lens protein is found as PMSO [15]. Age-related cataract, also called mature onset cataract, is an opacity of the eye lens that occurs relatively late in life, arising as a consequence of light scatter. Oxidation of lens proteins is a key event in cataractogenesis associated with loss of protein function, lens protein aggregation, protein proteolysis, and ultimately cataract formation [8] [16]–[17]. Age-related cataract is an extremely prevalent disease that is the leading cause of world blindness and the leading cost of Medicare surgery in the US [18]. At present, surgery is the only treatment for age-related cataract.

Unlike the majority of lens protein oxidations that are irreversible, PMSO formation is repairable by a unique family of enzymes called the methionine sulfoxide reductases (Msrs). Oxidation of methionine generates a 50∶50 mixture of S- and R-forms of PMSO as a consequence of sulfur oxidation [19]. The Msr family consists of a single enzyme, called MsrA, which specifically repairs the S-form of PMSO and three separate enzymes, called MsrB1, MsrB2 and MsrB3, which collectively recognize the R-form of PMSO. Thus, statistically, 50% of PMSO is repaired by MsrA while the remainder is repaired by one or more MsrBs. MsrA and the MsrBs have been shown to provide oxidative stress resistance to mammalian cells including eye lens cells [20]–[23]. Of the Msrs, MsrA is the best characterized. MsrA has been reported to extend lifespan by up to 70% through its over-expression in *Drosophila melanogaster* [24], while deletion of MsrA in mice was reported to decrease maximum lifespan by about 40% compared to wild type mice [25].

MsrA has been shown to play an important role in protection of lens cells against oxidative damage and it has been shown to be required for the maintenance of lens transparency *in vivo* [20]–[21] [26]–[27]. Gene silencing of MsrA decreases the resistance of lens epithelial cells to H_2_O_2_-induced oxidative stress resulting in increased mitochondrial ROS levels in human lens cells [20] and loss of lens cell mitochondrial function [21]. Deletion of the MsrA gene in mice leads to oxidative stress-induced cataract [26]. By contrast, over-expression of MsrA in human lens cells protects against oxidative stress and preserves mitochondrial function [20]. Recently, both cytochrome c (cyt c) [26] and α-crystallin/sHSP [27] have been identified as key targets of MsrA function in the lens. Both proteins are critical for lens function. Cyt c is essential for mitochondrial electron transfer and is a key initiator of apoptosis in mammalian cells [28]. α-crystallin/sHSP is a molecular chaperone that is essential for the maintenance of lens transparency whose deletion has been shown to result in cataract formation [29]–[33]. α-crystallin/sHSP is composed of two polypeptide subunits called αA- and αB-crystallin [30]. Analysis of MsrA knockout mouse lenses revealed oxidation of αB-crystallin/sHSP at methionine 68 [27]. Oxidation of α-crystallin/sHSP *in vitro* resulted in the specific oxidation of αA at methionine 138 and the specific oxidation of αB-crystallin at methionine 68 [27]. These oxidations resulted in a 50% loss of chaperone activity. Treatment of methionine oxidized α-crystallin/sHSP with MsrA in the presence of dithiothreitol (DTT) resulted in complete repair of the oxidized methionines and the restoration of α-crystallin/sHSP chaperone activity [27]. Further analysis of the MsrA knockout mouse lenses revealed oxidation of cyt c at met 80 [26]. *In vitro* analysis of met 80 oxidized cyt c showed loss of cyt c oxidase activity and gain of cyt c peroxidase activity [26]. Treatment of cyt c oxidized at met 80 with MsrA in the presence of DTT resulted in repair of oxidized met 80, restoration of cyt c oxidase activity, and decreased cyt c peroxidase activity [26]. In all of these studies DTT was used as an MsrA reducing system since the actual lens reducing system is not known. Since MsrA knockout mice exposed to hyperbaric oxygen develop late-onset cataract in association with methionine oxidation of essential lens proteins, understanding the reducing requirements for MsrA action on these proteins in the lens is an essential step towards understanding the etiology of human cataract and the potential development of therapies to prevent cataract development and possibly other oxidative stress associated diseases.

MsrA repair of PMSO proceeds in three steps. First, nucleophilic attack by the catalytic cysteine (Cys 51) on the target PMSO releases 1 mol of protein methionine per mol of enzyme and results in the transient formation of a sulfenic acid intermediate within the MsrA molecule. The recycling cysteine (Cys 198) then attacks the sulfenic acid intermediate forming an intramonomeric disulfide bond with the concomitant release of 1 mol H_2_O. A key step in this reaction is reduction of the intramonomeric disulfide bond making the action of MsrA dependent on the availability of a reducing system to catalytically reactivate the enzyme [34]. In *E.coli* and *Saccharomyces cerevisiae* MsrA was shown to be able to use thioredoxin (Trx) as its reducing system [35]–[36]. More recently, it has been shown that thionein could also serve as a reducing system for *E.coli* MsrA and human MsrB3 [37], suggesting that multiple reducing components can be utilized for MsrA activity. Given the important role for MsrA in lens function and the maintenance of key lens proteins, we sought to identify additional lens reducing systems for the MsrA function.

One novel protein that could serve as a reducing agent for MsrA repair activity is thioredoxin-like 6 protein (TXNL6), which is called rod derived cone viability factor (RdCVF) in mice. TXNL6 has recently been discovered to confer viability to retinal cones [38] and its deletion shown to decrease resistance to oxidative stress in retinal cells of mice [39]. TXNL6 shares the catalytic CXXC domain of Trx as well as the characteristic thioredoxin fold [39]. Based on the recent report suggesting that thionein in addition to Trx could serve to activate MsrA activity, we hypothesized that TXNL6 might act as a reducing system for MsrA repair of the essential lens proteins α-crystallin/sHSP and cyt c. We also hypothesized that altered levels of TXNL6 upon oxidative stress exposure of lens cells could augment the ability of MsrA to repair essential lens proteins under oxidative conditions and regulate MsrA activity through competition between different reducing systems.

Our results indicate that in addition to the retina [38], TXNL6 is expressed in the human eye lens and multiple other human tissues, that TXNL6 co-localizes with MsrA in the cytosol and the mitochondria of lens cells, that TXNL6 expression is inducible in lens cells upon oxidative stress exposure, and most importantly, that TXNL6 acts as a novel reducing system for MsrA repair of cyt c and α-crystallin/sHSP. These findings provide evidence that TXNL6 is critical for the action of MsrA under oxidative stress conditions and that TXNL6 is likely to play an important role in cataract formation through its activation of MsrA.

## Methods

### Lens cell culture

A human lens epithelial cell line (HLE-B3) immortalized by infection with adenovirus 12-SV40 [40] was grown and cultured in DMEM (Invitrogen, Carlsbad, CA) supplemented with 15% FBS (Invitrogen), gentamicin (50 units/ml; Invitrogen), penicillin-streptomycin antibiotic mix (50 units/ml; Invitrogen) and fungizone (5 ul/ml; Invitrogen) at 37°C in the presence of 5% CO_2_.

### Isolation of cytosolic and mitochondrial proteins

HLE-B3 cells (harvested at approximately passage 15) were washed once in PBS before freezing at −80°C to weaken the cell membrane. Pellets were frozen and thawed prior to re-suspension in Clami buffer (250 mM sucrose, 1 mM EDTA, 70 mM KCl in PBS) and lysed by Dounce homogenization. Lysates were centrifuged at 700 g for 10 min at 4°C, the supernatant was collected and centrifuged at 10,000 g for 15 min at 4°C. The resulting supernatant contained the cytosolic protein. The pellet containing the mitochondria was resuspended in 100 µl of universal buffer (50 mM Tris-HCl pH 7.4, 150 mM NaCl, 2 mM EDTA, 2 mM EGTA, 0.2% Triton X-100, 0.3% NP-40), and incubated on ice for 30 min. The mixture was centrifuged at 20,000 g for 30 min at 4°C. The supernatant containing the mitochondrial protein was stored at −20°C until used. Protein concentrations were determined by Bradford protein assay as previously described [20].

### Analysis of TXNL6, Trx 1 and Trx 2 transcripts in human tissues

TXNL6, Trx 1, Trx 2 and GAPDH transcripts were evaluated by semi-quantitative RT-PCR using the SuperScript® III one-step RT-PCR system with Platinum Taq polymerase (Invitrogen) according to the manufacturer's instructions. 200 ng of total RNA was assayed from indicated human tissues. RNA was isolated from microdissected human lens epithelium and fiber cells as previously described [41]. Lens epithelium was separated off and the cortical and nuclear fibers harvested. The average age of clear lenses obtained from the West Virginia Eye Bank was 63 years of age. All lenses were obtained within 24 h post mortem. RNA from other tissues was purchased from BD Biosciences (Franklin Lakes, NJ). GAPDH was amplified as the internal control transcript for 30 PCR cycles with a 60°C annealing temperature using forward primer – CCACCCATGGCAAATTCCATGGCA and reverse primer - TCTAGACGGCAGGTCAGGTCCACC. TXNL6 transcripts were amplified for 35 PCR cycles with a 59°C annealing temperature and the primer sequences: Forward primer – GCCGCATCCTGATCCGCAACAATA and reverse primer – CACTGAGAACTGGCGCCCGAGGTC. Trx 1 and Trx 2 transcripts were amplified for 35 cycles with annealing temperatures of 55°C and primer sequences Trx 1 – forward primer – CCTTTCTTTCATTCCCTCTCTGA and reverse primer – GCAACATCCTGACAGTCATCCA, Trx 2 - forward primer – CTGGTGGCCTGACTGTAACAC and reverse primer – GTTGACCACTCGGTCTTGAAA.

### Localization of TXNL6 to the mitochondria in human lens cells

HLE-B3 lens cells were plated onto coverslips and incubated overnight in complete media. Double immunofluoresence-simultaneous staining was conducted by fixing cells with 3.7% formaldehyde in PBS, blocking with 1% BSA and permeabilizing with 0.25% Triton X-100 in PBS. For co-localization of TXNL6, Trx 1 and Trx 2 to the mitochondria, first HLE cells were stained with Mitotracker Red CMXRos (Molecular Probes, Invitrogen) for 45 min at 250 nM as indicated by the manufacturer's protocol. Following mitotracker red staining, rabbit polyclonal anti-TXNL6 (ProteinTech Group Inc, Chicago, IL) at 1∶1000 or rabbit polyclonal anti-Trx 1 at 1∶1500 (Abcam, Cambridge, MA), or primary anti-Trx 2 (ProteinTech Group Inc) at 1∶1000 were incubated overnight in 4°C. Cells were washed three times with PBS, and subsequently incubated with Alexa Fluor 488 goat anti-rabbit secondary (Invitrogen) for 1 h at room temperature at 1∶1000 dilutions. HLE cells were washed with three times with PBS, and mounted onto slides. Immunofluoresence staining was visualized with a Zeiss LSM 700 Confocal microscope.

### Preparation, purification of and activation of TXNL6

TXNL6 protein was expressed from the Origene (Rockville, MD) Homo sapiens TXNL6 (nucleoredoxin-like 1, NXNL1 gene) vector which contains the TXNL6 cDNA under the control of the T7 promoter. TXNL6 protein was produced from the TXNL6 vector using the T7 promoter-based Quick TNT coupled transcription/translation system according to the instructions and conditions specified by the manufacturer (Promega, Madison, WI). Crude protein preparations were subsequently purified by gel filtration on a Superdex™ 200 10/300 column. The resulting TXNL6 protein was examined by SDS-PAGE and western blot analysis using the ProteinTech rabbit polyclonal TXNL6-specific antibody for purity and identity. TXNL6 protein was activated by reduction using a 5 molar excess of DTT (Sigma) and incubating for 30 min at room temperature in Tris-HCl pH 7.4. DTT was removed by ultrafiltration using an Amicon stirred ultrafiltration cell with a 1000 kDa nominal molecular weight limit (NMWL) membrane.

### Analysis of TXNL6 transcript and protein in human lens cells under oxidative stress conditions

HLE-B3 cells were plated in 6 well plates at a density of 4×10^5^ cells per well. Cells were incubated in serum free medium for 2 h and then exposed to 200 µM H_2_O_2_ (or PBS as a control) for 2 h in serum free medium as previously described [42]. Cells were washed once in PBS and returned to serum-supplemented medium (15% serum) for recovery. Cells were harvested at the times indicated and processed for RNA and protein. For protein isolation, cells were washed with ice cold PBS and directly lysed on the plate in lysis buffer (50 mM Tris-HCl ph 7.4, 150 mM NaCl, 1 mM EDTA, 1% NP40, 0.25% sodium deoxycholate) containing 1∶1000 protease inhibitor cocktail (Sigma-Aldrich, St Louis, MO). The lysates were sonicated for 10 sec×2 and centrifuged at 10,000 g for 10 min; the supernatant contained the total cell extract and was stored at −20°C until used. For RNA isolation Trizol® reagent (Invitrogen) was used. Briefly, cells were washed in 1 ml of ice cold PBS, 1 ml of Trizol® reagent was added to the plate and cells harvested using a cell scrapper. Phase separation was performed by adding 0.2 ml of chloroform, vortexing for 15 sec and incubating at room temperature for 3 min. The mixture was then centrifuged at 12,000 g for 15 min at 4°C. The supernatant was removed into a fresh tube and precipitation of RNA was achieved by adding 0.5 ml of isopropyl alcohol and incubating at room temperature for 10 min. The precipitate was centrifuged at 12,000 g for 10 min at 4°C and the resulting RNA pellet washed twice in 75% alcohol. The washed pellet was air dried and resuspended in RNAase/DNAase free water. RNA was stored at −20°C until used. TXNL6, Trx1, Trx 2 and GAPDH transcript levels were analyzed by semi-quantitative RT-PCR as described above. TXNL6 protein levels were analyzed by western blot analysis as described below.

### SDS-PAGE and western blotting

SDS-PAGE and western blotting were carried out as previously described [26], western blots were developed using ECL™ chemiluminescence reagents (GElifesciences, Buckinghamshire, UK). Protein samples were mixed with 2x sample buffer at a 1∶1 volume ratio and heated at 100°C for 5 min. Samples were separated by electrophoresis on either 15% SDS-PAGE gels (microdissected lens fractions, whole lens proteins, and HLE-B3 protein extracts) or 16% Tricine-SDS-PAGE gels for CNBr cleaved proteins at room temperature for 1.5 h at 120 volts. Tricine-SDS-PAGE gels were used for more complete separation of low molecular weight CNBr products of cyt c and α-crystallin. For Tricine-SDS-PAGE gel electrophoresis the method of Schagger, 2006 [43] was used.

The following antibody concentrations were used where appropriate: primary anti-TXNL6 (ProteinTech) 1∶1000, primary anti-Trx 1 at 1∶1000 (Abcam), primary anti-Trx 2 (ProteinTech) 1∶1000, primary anti-MsrA (Abcam) 1∶2000, and secondary anti-rabbit 1∶5000. Gels were also stained with Colloidal blue for 4 h and de-stained overnight in ddH_2_O.

### Cytochrome c peroxidase assay

Cyt c (horse heart, Sigma-Aldrich) was treated with hypochlorous acid (HOCl, Sigma-Aldrich) and subsequently MsrA/TXNL6 as follows: cyt c was incubated with HOCl in a 4∶1 ratio for 15 min at room temperature. After incubation, the excess HOCl was removed from the oxidized cyt c using ultrafiltration on an Amicon cell. Oxidized cyt c (291 µM) was incubated with MsrA (1.9 µM) for 2 h at 37°C in the presence of TXNL6 (1.39 µM) with rat liver thioredoxin reductase (TxrR)(2.4 µg, Sigma-Aldrich) and NADPH (500 µM, Sigma-Aldrich). Repair of oxidized cyt c was also carried out using human Trx 1 (1.39 µM, Sigma-Aldrich) with TxrR/NADPH or yeast Trx 2 (1.39 µM, Abcam) with the same reducing system. As controls oxidized cyt c was incubated with MsrA with no reducing system, MsrA and TXNL6 in the absence of TxrR/NADPH, and TXNL6 with TxrR/NADPH but no MsrA. The enzymatic peroxidase activity was measured by 2,2′-azino-bis(3-ethylbenzothiazoline-6-sulfonic acid)diammonium salt (ABTS) oxidation. The effect of oxidation on the peroxidatic activity of cyt c was assayed by adding 0.6 µM cyt c to 1 ml of the mixture containing 1.3 mM ABTS, 12 mM H_2_O_2_, and 100 µM diethylenetriaminepentaacetic acid in 100 mM phosphate buffer (pH 7.4) at 20°C, followed by measuring the increase in absorbance at 420 nm [44]. Results from the cytochrome c peroxidase assay are expressed as means ± standard deviation (SD). Differences among treatments of oxidized cyt c were determined using Tukey's test following one-way ANOVA. p<0.05 was considered statistically significant.

### Analysis of methionine oxidized and MsrA-repaired α-crystallin and cytochrome c

Oxidation of α-crystallin/sHSP and cyt c and MsrA repair of oxidized mets in these proteins was analyzed as previously described [26]–[27] except that TxrR/NADPH and either TXNL6, Trx1 or Trx 2 were used instead of DTT as the reducing system for MsrA. Cyanogen bromide (CNBr) hydrolyzes peptide bonds at the C-terminus of met residues; it does not cleave mets converted to PMSO and is therefore a sensitive detection method to identify met sulfoxide in proteins [45]. For α-crystallin/sHSP, bovine total α-crystallin (1.0 mg, Sigma-Aldrich) was incubated with HOCl (909 µM; molar ratio 100∶1) at room temperature for 15 min to convert mets to met sulfoxide as previously described [27]. Excess HOCl was removed from the oxidized α-crystallin using ultrafiltration on an Amicon cell. Oxidized mets in α-crystallin/sHSP were repaired by incubating oxidized α-crystallin/sHSP (6.36 µM) with MsrA (100 nM) using either DTT (15 mM) or TXNL6 (10 µg; final concentration 2 µM and 20 µg; final concentration 4.17 µM) with TxrR/NADPH as the reducing system for the MsrA enzyme. Horse heart cyt c protein (Sigma-Aldrich) (0.2 mM) was incubated with HOCl at a 4∶1 (0.8 mM) molar ratio at room temperature for 15 min. For repair of oxidized mets, oxidized cyt c (291 µM) was incubated with 1.9 µM MsrA for 2 h at 37°C using either DTT (15 mM) or TXNL6 (10 µg; final concentration 1.39 µM) with TxrR/NADPH as the reducing system for the MsrA enzyme. Purified mouse MsrA was obtained as a gift from Dr. Rodney Levine (NIH).

Samples were dried on a vacuum centrifuge and then treated or not treated by CNBr cleavage. Treated or untreated α-crystallin/sHSP or cyt c was then diluted in 70% formic acid to 10–20 mg/ml as required by the standard CNBr protocol [45]. CNBr (Sigma-Aldrich) was added in a 2∶1 w/w ratio and the reaction mixture incubated at room temperature for 20 h in the dark. The reaction was terminated by addition of 5 volumes ddH_2_O and 5 volumes 1 M ammonium bicarbonate. Samples were then concentrated using an Amicon stirred ultrafiltration cell. 5 µg of treated or untreated α-crystallin/sHSP protein or cyt c was run on a 16% Tricine-SDS-PAGE gel at 90 mV. The gels were fixed in 40% methanol, 10% acetic acid for 2 h and stained overnight in Coomassie blue stain. Gels were de-stained in 50% methanol, 10% acetic acid.

## Results

### TXNL6 is expressed in the eye lens and other human tissues

Previous work in mice suggested that expression of TXNL6 (known as RdCVF in mice) is retina-specific; however, expression in the lens or other ocular tissues was not reported [38]. Here, TXNL6 levels were examined in two separate lens cell sub-types called the lens epithelium and the lens fibers. The lens epithelium covers the anterior surface of the organ, and the lens fiber cells lie directly beneath the lens epithelium [46]. The lens epithelial cells contain intact organelles and the majority of transporters and metabolic enzymes in the lens [47]–[48], while the fiber cells are for the most part devoid of organelles and contain the majority of structural crystallin proteins required for light refraction and lens transparency [49]. The lens is a unique organ since the cells and proteins contained in the lens fibers must remain functional for the life of the individual [46]. Protein aggregation in the lens fibers as a result of oxidation, protein modifications, and/or proteolysis results in loss of lens transparency and cataract formation [7] [50]. Thus, expression of TXNL6 in the lens epithelium could suggest a mitochondrial, transport or other epithelial specific function for TXNL6, while expression in the lens fibers could suggest a protective and/or repair function for TXNL6 in the maintenance of lens crystallins or other lens fiber proteins.

To examine the possible expression of TXNL6 in lens epithelial and lens fibers, whole human lenses (average age of clear lenses obtained was 63 years of age) were microdissected into epithelium and cortical fiber cells and RNA and protein prepared. TXNL6 transcripts were monitored by RT-PCR and TXNL6 protein examined by western analysis using a TXNL6-specific antibody. Trx 1 and Trx 2 transcripts were also examined as indicated for comparison since these proteins are known to be expressed in the lens [51] and could also function as a reducing system for lens MsrA.

**Figure 1:**
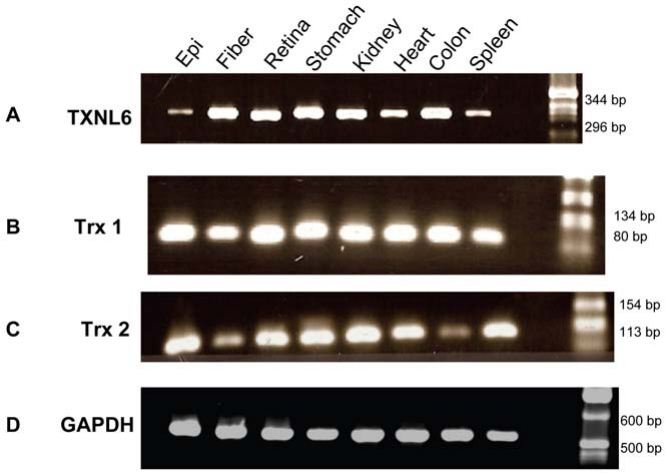
TXNL6 transcript is detected in the human lens epithelium and lens fiber cells and in other human tissues. Ethidium bromide stained agarose gels show Panel A - TXNL6, Panel B - Trx 1, Panel C - Trx 2 and Panel D GAPDH (control) transcript from 200 ng of RNA obtained from human tissues - 1. Lens epithelium, 2. Lens Fiber, 3. Retina, 4. Stomach, 5. Kidney, 6.Heart, 7. Colon, and 8. Spleen.

**Figure 2:**
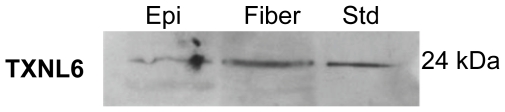
TXNL6 protein is present in human lens epithelium and fiber cells. SDS-PAGE and immunoblotting of microdissected lens epithelium (Epi) and lens fiber cell total protein extracts (20 µg) with a TXNL6-specific antibody.

TXNL6 transcript was detected at significant levels in lens fiber cells with lower levels detected in the lens epithelium (Figure 1 Panel A). Similarly, Trx 1 was ubiquitously expressed in the lens (Figure 1 Panel B), while Trx 2 expression was mainly localized to the lens epithelium as expected based on its known mitochondrial expression pattern (Figure 1, Panel C). As a control for RNA integrity and for loading, GAPDH was expressed equally in all tissues (Figure 1 Panel D). TXNL6 (Figure 1 Panel A), Trx 1 (Figure 1 Panel B) and Trx 2 (Figure 1, Panel C) transcripts were also detected in human retina, stomach, kidney, heart, colon, and spleen. Interestingly, the levels of TXNL6 transcript appeared higher in the lens fiber cells than in lens epithelial cells. Similar patterns for TXNL6 protein expression were identified for lens epithelial and fibers by western blot analysis (Figure 2). These results demonstrated that TXNL6 is expressed in the lens epithelium, lens fiber cells and other human tissues. They also indicated that TXNL6 could have a significant function in both lens epithelial and fiber cells.

### TXNL6 is detected in the cytosol and the mitochondria of lens cells

MsrA has been localized to the cytosol and mitochondria of rat [52] and mouse cells [53]. To determine if TXNL6 might co-localize with MsrA in lens cells, we compared the sub-cellular localization pattern of TXNL6 in lens cells first by western blotting of lens cytosolic and mitochondrial protein fractions and second by immunofluoresence staining. We also examined the sub-cellular localization pattern of Trx 1 and Trx 2 as controls, since Trx 2 is known to exhibit a mitochondrial specific expression pattern [54] and Trx 1 is know to be mainly cytosolic however movements within the cellular compartments are known [54] [55].

**Figure 3:**
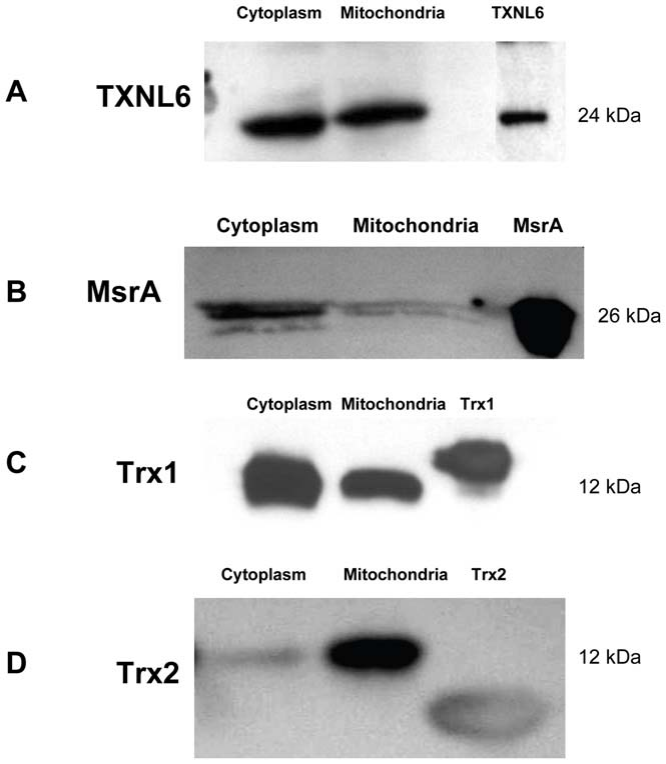
TXNL6 is present in protein extracts prepared from the cytoplasm and mitochondria of human lens epithelial cells. SDS-PAGE and immunoblotting of Panel A - TXNL6, Panel B - MsrA, Panel C - Trx 1, and Panel D -Trx 2 in 5 µg of protein extracts from the cytoplasm and mitochondria of HLE-B3 lens epithelial cells using specific antibodies.

Western blot analysis of cytosolic and mitochondrial protein extracts from HLE-B3 cultured lens epithelial cells suggested that both TXNL6 and MsrA are located in both the cytosol and the mitochondria of lens cells (Figure 3, Panel A and B).

**Figure 4:**
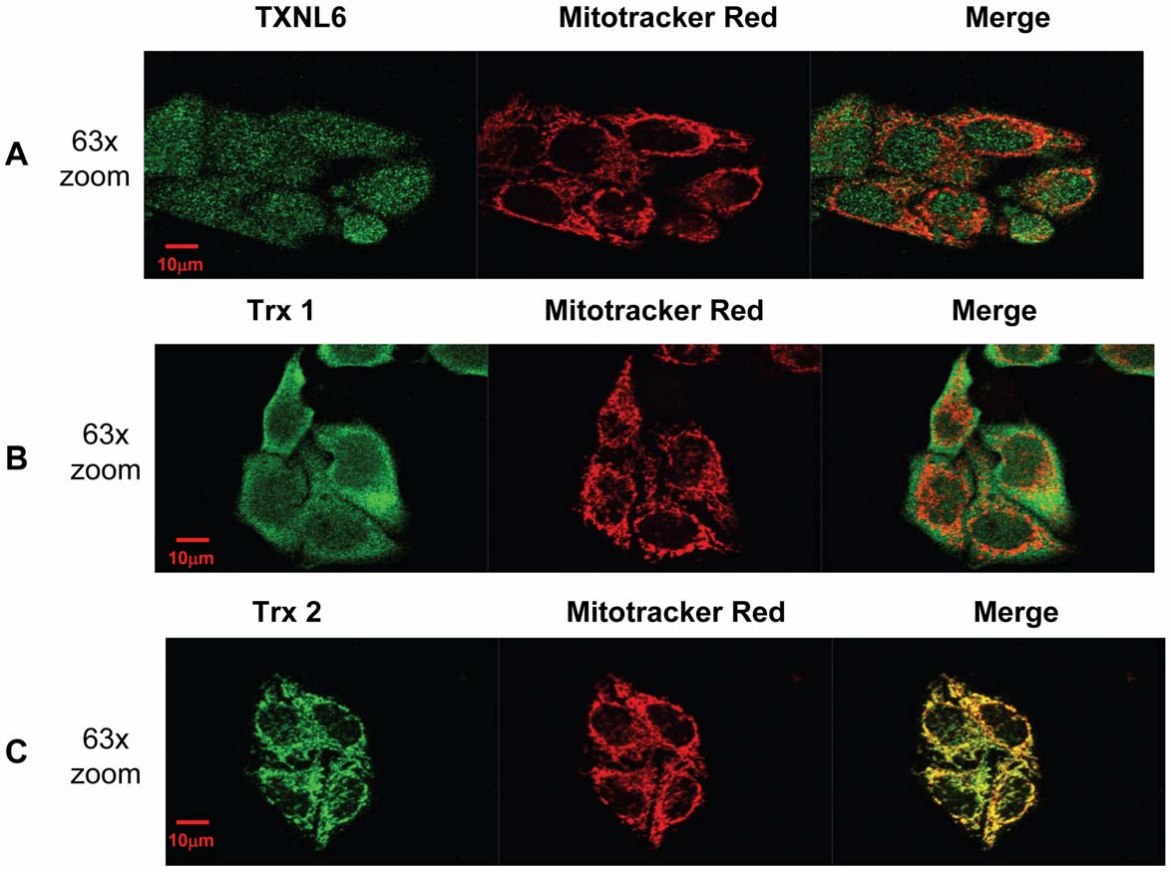
Mitochondrial and cytosolic localization of TXNL6 in human lens epithelial cells. Co-localization of Panel A - TXNL6 (green), Panel B - Trx 1 (green) and Panel C - Trx 2 (green), mitotracker red – a specific mitochondrial marker and merging of the two images (orange/yellow) in HLE-B3 lens epithelial cells by immunofluoresence microscopy.

As controls, Trx 1 was located in both the cytosol and mitochondria (Figure 3, Panel C) of the lens cells, while Trx 2 was localized to the mitochondria as expected (Figure 3, Panel D) demonstrating minimal contamination of the mitochondrial extracts with cytosolic components. A secondary examination of TXNL6 expression in HLE cells was conducted *in vivo* by immunofluoresence staining (Figure 4, Panel A), TXNL6 was co-localized with the mitochondrial-specific marker Mitotracker Red along with Trx 1 and Trx 2 as controls. Consistent with the western blot analysis, TXNL6 (green) co-localized with mitotracker (red) in the cultured HLE-B3 human lens epithelial cells (Figure 4, Panel A - orange). Previous immunofluoresence staining of the HLE-B3 cells with an MsrA-specific antibody [26] revealed an identical expression pattern for MsrA in these cells. As controls, immunofluoresence staining indicated that Trx 1 is located in both the cytosol and mitochondria of these cells (Figure 4, Panel B) while Trx 2 is located specifically in the mitochondria of these cells as expected (Figure 4, Panel C) and in agreement with the western blot analysis. These data provide strong evidence that TXNL6 co-localizes with MsrA [26] in both the cytoplasm and mitochondria of lens epithelial cells.

### Oxidative stress induces TXNL6 mRNA and protein in HLE-B3 cells

**Figure 5:**
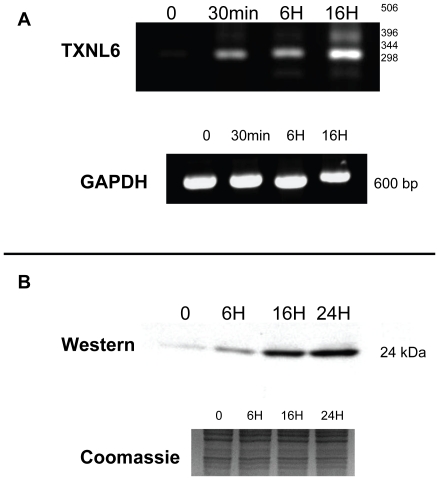
TXNL6 mRNA and protein are induced by oxidative stress exposure of human lens epithelial cells. **A.** Ethidium bromide stained agarose gels showing TXNL6 and control GAPDH transcript levels in 200 ng RNA isolated from HLE-B3 human lens epithelial cells at 0, 30 min, 6 h and 16 h recovery following a 2 h exposure to 200 µM H_2_O_2_. **B.** Western blot showing TXNL6 protein levels in 5 µg of total protein extract isolated from HLE-B3 lens epithelial cells at 0, 6 h, 16 h and 24 h recovery following a 2 h exposure to 200 µM H_2_O_2_. The coomassie blue stained SDS-PAGE gel is shown as a control for equal protein loading.

Significant evidence suggests that oxidative stress exposure and accumulation of oxidized lens components upon aging is a major factor in cataract development [8] [16] [56] [17] and likely accounts for increased PMSO formation associated with lens aging and cataract. Since MsrA protects lens cells against oxidative stress, we hypothesized that any reducing system needed for MsrA activity might be activated by oxidative stress and thereby augment the activity of MsrA to defend lens cells under these conditions. To examine the levels of TXNL6 under oxidative stress conditions, HLE-B3 lens epithelial cells were treated with 200 µM H_2_O_2_ for 2 hours as previously established for optimal expression of oxidative stress-specific lens transcripts [42] and the levels of TXNL6 were examined at the RNA and protein level (Figure 5). H_2_O_2_-treatment led to an increase in TXNL6 transcript levels over the 16 h post-treatment time interval (Figure 5, Panel A). Corresponding TXNL6 protein levels were also analyzed by western analysis using a TXNL6-specific antibody. TXNL6 protein levels significantly increased over a 24 h period following treatment (Figure 5, Panel B). Coomassie staining of the SDS-PAGE gel indicates equal loading of total cellular protein (Figure 5). As a control, no increase in TXNL6 expression was seen in untreated HLE-B3 cells maintained identically (data not shown). These data demonstrate that TXNL6 is induced in lens epithelial cells upon oxidative stress exposure and suggest that TXNL6 expression is a direct response of lens cells to oxidation.

### TXNL6 can serve as reducing system for MsrA repair of oxidized α-crystallin/sHSP and cytochrome c

MsrA has previously been shown to be required for the repair and preservation of the function of the essential lens proteins cyt c [26] and α-crystallin/sHSP [27] which regulate lens epithelial cell apoptosis [28] [33] and the maintenance of lens crystallin structure [32]. These previous studies used HOCl as an oxidant since it preferentially targets met residues in proteins [57]. Treatment of cyt c with HOCl results in the specific oxidation of met 80 to met 80 sulfoxide which results in loss of cyt c function [26]. α-crystallin/sHSP consists of two protein subunits called αA- and αB-crystallin [30]. Treatment of α-crystallin/sHSP with HOCl results in the specific oxidation of met 138 in αA- and met 68 of αB-crystallin/sHSP [27]. To date, only DTT has been used as a reducing system for MsrA repair of cyt c and α-crystallin/sHSP *in vitro* [26]–[27]. To determine if TXNL6 could act as a reducing agent for MsrA, we prepared cyt c specifically oxidized at met 80 [26] and α-crystallin/sHSP specifically oxidized at met 138 of αA-crystallin and met 68 of αB-crystallin [27] and determined the ability of MsrA to repair these using TXNL6 as a reducing system for MsrA activity.

**Figure 6:**
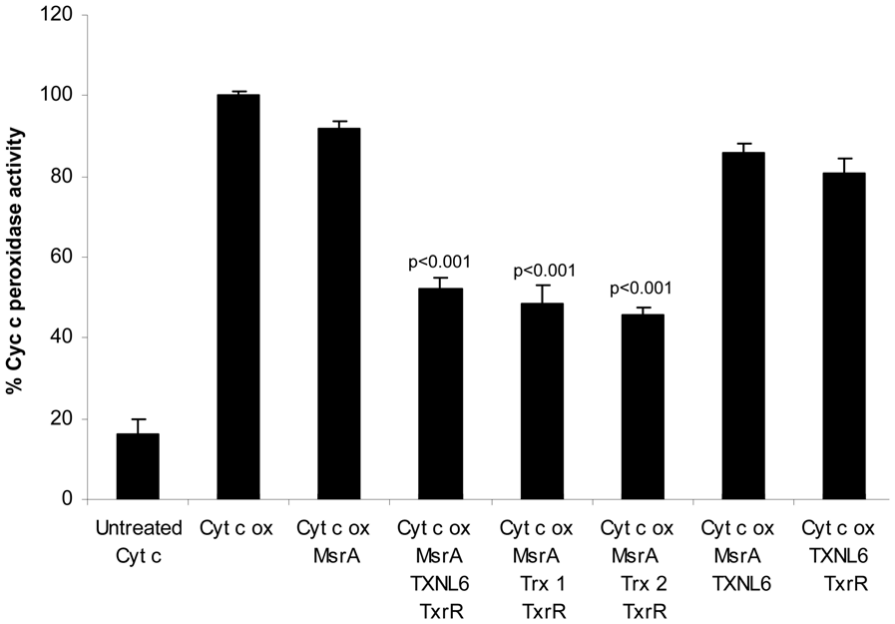
TXNL6-mediated MsrA repair of cytochrome c inhibits cytochrome c peroxidase activity. Representative graph (from 3 independent experiments) of cytochrome c (cyt c) mediated peroxidase activity. The graph shows mean values for an N of 3 ± SD. Cyt c oxidized (cyt c ox) (incubated for 15 min with a 4∶1 molar ratio of HOCl) is 100% peroxidase activity, all other activities are expressed as a percentage of the cyt c ox activity. The untreated cyt c protein has 16% cyt c peroxidase activity compared to cyt c ox. Cyt c ox incubated with MsrA in the absence of a reducing system has 91% activity of the oxidized. Treatment of the cyt c ox (291 µM) with MsrA (1.9 µM for 2 h at 37°C) and TXNL6 (1.39 µM) with thioredoxin reductase (TxrR) and NADPH leads to a statistically significant (p<0.001) 48% decrease in peroxidase activity. Similarly treatment of cyt c ox with MsrA and either Trx 1 or Trx 2 (both 1.39 µM) with TxrR/NADPH also significantly (p<0.001) decreased both peroxidase activities by 52% and 54% respectively. Incubation of cyt c ox with MsrA and TXNL6 in the absence of TxrR/NADPH resulted in just a 14% decrease in cyt c peroxidase activity while incubation of cyt c ox with TXNL6 and TxrR/NADPH in the absence of MsrA resulted in 19% decrease in cyt c peroxidase activity. p values were obtained using Tukey's test following one-way ANOVA.

A key characteristic of met 80 oxidized cyt c is conversion of cyt c into an active peroxidase involved in the initiation of apoptosis [26] [58]–[60]. Thus, cyt c peroxidase activity is a sensitive assay for cyt c met 80 oxidation. As a first step in examining the ability of TXNL6 to act as a reducing system for MsrA repair of cyt c, we examined the relative peroxidase activities of untreated (met 80 reduced), met 80 oxidized and met 80 oxidized cyt c that was repaired by MsrA using TXNL6 with TxrR/NADPH as a reducing system (Figure 6) and Trx 1 or Trx 2 with TxrR/NADPH as the reducing system (Figure 6). As controls, met 80 oxidized cyt c was incubated with MsrA with no reducing system, with MsrA and TXNL6 in the absence of TxrR/NADPH and finally TXNL6 with TxrR/NADPH in the absence of MsrA. As expected, met 80 oxidation of cyt c resulted in a statistically significant increase (p<0.001) in peroxidase activity compared to the untreated protein (Figure 6). The peroxidase activity of met 80 oxidized cyt c is labeled as 100% peroxidase activity in these studies and all activities are relative to met 80 oxidized cyt c. Untreated cyt c has only 15% peroxidase activity by comparison (Figure 6). Treatment of met 80 oxidized cyt c with MsrA using TXNL6 and TxrR/NADPH as a reducing system significantly (p<0.001) decreased cyt c peroxidase activity by approximately 50% suggesting that TXNL6 was able to serve as a reducing system for MsrA repair of met 80 oxidized cyt c (Figure 6). In addition, repair of met 80 oxidized cyt c using either Trx 1 or Trx 2 and TxrR/NADPH as the reducing systems also resulted in a significant (p<0.001) approximate 50% reduction in peroxidase activity for both reducing systems. As a control, repair using MsrA in the absence of a reducing system resulted in only a 9% reduction in peroxidase activity. Interestingly, repair of met 80 oxidized cyt c using MsrA and TXNL6 in the absence of TxrR/NADPH results in only a 14% decrease in peroxidase activity indicating that TXNL6 must be reduced by TxrR in order to activate the MsrA enzyme and that TXNL6 can use TxrR/NADPH as its reducing enzyme. In the absence of MsrA, incubation of met 80 oxidized cyt c with TXNL6 and TxrR/NADPH resulted in a 19% decrease in peroxidase activity, demonstrating that cyt c met 80 repair is dependent on a functional MsrA.

**Figure 7:**
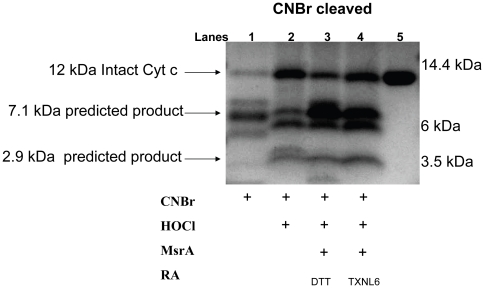
MsrA repairs oxidized methionines in cytochrome c using TXNL6 as a reducing agent. Coomassie staining of a Tricine-SDS-PAGE gel following CNBr cleavage of oxidized cyt c (5 µg). Lane 1 untreated cyt c cleaved with CNBr. Lane 2 Oxidized cyt c (0.2 mM oxidized with 0.8 mM HOCl; a 4∶1 Molar ratio) cleaved with CNBr. Lane 3 Oxidized cyt c (291 µM) treated with MsrA (1.9 µM) and DTT (15 mM) for 2 h at 37°C and cleaved with CNBr. Lane 4 Oxidized cyt c (291 µM) treated with MsrA (1.9 µM) and TXNL6 (10 µg; 1.39 µM) for 2 h at 37°C and cleaved with CNBr. RA - reducing agent/system.

**Figure 8:**
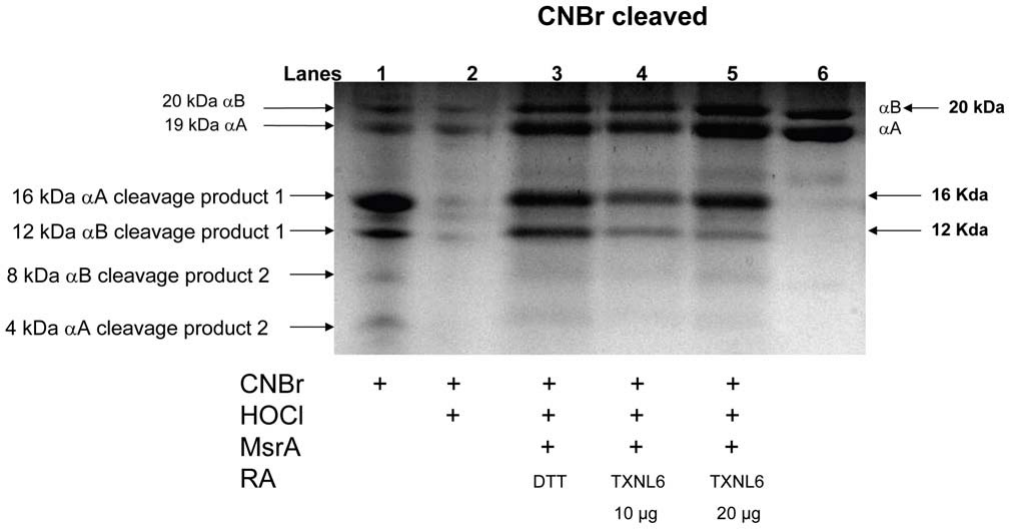
MsrA repairs oxidized methionines in α-crystallin using TXNL6 as a reducing agent. Coomassie staining of a Tricine-SDS-PAGE gel following CNBr cleavage of oxidized α-crystallin (5 µg). Lane 1 untreated α-crystallin cleaved with CNBr. Lane 2 Oxidized α-crystallin (9.09 µM oxidized with 909 µm HOCl; a 100∶1 Molar ratio) cleaved with CNBr. Lane 3 Oxidized α-crystallin (6.4 µM) treated with MsrA (100 nM) and DTT (15 mM) for 2 h at 37°C and cleaved with CNBr. Lane 4 Oxidized α-crystallin (6.4 µM) treated with MsrA (100 nM) and TXNL6 (10 µg; 1.39 µM) for 2 h at 37°C and cleaved with CNBr. Lane 5 Oxidized α-crystallin (6.4 µM) treated with MsrA (100 nM) and TXNL6 (20 µg; 2.78 µM) for 2 h at 37°C and cleaved with CNBr. RA - reducing agent/system.

To further demonstrate that TXNL6 can serve as a reducing system for MsrA repair of cyt c, untreated cyt c and met oxidized cyt c and met oxidized α-crystallin/sHSP were treated with MsrA and TXNL6 with TxrR/NADPH and the samples analyzed by Tricine-SDS-PAGE following CNBr cleavage (Figures 7 and 8). MsrA with DTT as the reducing system was examined as a control. Cleavage of un-oxidized cyt c with CNBr should result in three peptides of molecular weights, 7.1 kDa, 2.9 kDa, and 1.8 kDa. If both mets in cyt c are oxidized, no CNBr products should be visualized. CNBr cleavage at met 80 should result in a 2.9 kDa band. CNBr cleavage of cyt c with oxidation at met 80 in the absence of oxidation at met 65 should result in 7.2 kDa and 4.5 kDa peptides; while oxidation of met 65 in the absence of oxidation at met 80 would yield 8.9 kDa and 2.9 kDa peptides.

As expected, CNBr cleavage of untreated cyt c resulted in the visualization of a 7.1 kDa peptide on the coomassie stained Tricine-SDS-PAGE gel as well as a lightly stained parent peptide at 12 kDa (Figure 7, Lane 1). The 1.8 kDa peptide was not visible in this gel. Oxidation of cyt c with a 4∶1 molar ratio of HOCl resulted in the loss of the 7.1 kDa peptide and an increase in the 12 kDa intact cyt c peptide (Figure 7, Lane 2). Incubation of the met oxidized cyt c with MsrA and TXNL6 repaired the oxidized mets in cyt c as evidenced by a significant increase in the staining intensity of the 7.1 and 2.9 kDa bands, and a decreased staining intensity of the intact cyt c 12 kDa peptide (Figure 7, Lane 4) compared to the oxidized cyt c (compare Figure 7 Lanes 2 and 4). As a control, oxidized cyt c was also repaired by MsrA using DTT as a reducing agent (Figure 7, Lane 3) [26]. The repair of cyt c by MsrA with TXNL6 (TxrR/NADPH) as a reducing system was not 100% efficient since a 50∶50 ratio of S- and R-epimers of PMSO are expected following reaction with HOCl and since MsrA is specific for the S-form of PMSO.

The ability of TXNL6 to serve as a reducing system for MsrA-repair of α-crystallin/sHSP was also evaluated using CNBr. Tricine-SDS-PAGE gel analysis of CNBr-cleaved untreated α-crystallin/sHSP (Figure 8, Lane 1) produced 6 peptide products, the original parent αA-crystallin (19 kDa) and αB-crystallin (20 kDa) peptides and four CNBr-cleavage products with molecular weights of approximately 16 kDa, 12 kDa, 8 kDa and 4 kDa. These bands are consistent with CNBr cleavage at met 138 of αA- and met 68 of αB-crystallin, respectively. The two smaller predicted cleavage products of approximately 4 kDa (αA-crystallin) and approximately 8 kDa (αB-crystallin) were also detected. Met-oxidation of α-crystallin (9.09 µM) with HOCl (0.9 mM for 15 min at room temperature; 100∶1 molar ratio) led to loss of cleavage by CNBr indicating the presence of oxidized met 68 of αB-crystallin and met 138 of αA-crystallin resulting in the detection of only αA-crystallin and αB-crystallin parent proteins (Figure 8, Lane 2). Incubation of the met-oxidized α-crystallin (6.36 µM) with MsrA (100 nM for 2 h at 37°C) and increasing amounts (10 µg or 20 µg) of TXNL6 with TxrR/NADPH as an MsrA reducing system repaired the oxidized mets as evidenced by the detection of cleavage products missing in the oxidized form (Figure 8, Lanes 4 and 5). As a control, DTT (15 mM) also resulted in repair of oxidized α-crystallin/sHSP (Figure 8, Lane 3) as previously shown [27]. Based on the intensity of the bands in the repaired samples (compare Figure 8, Lanes 1, 3 and 5) the repair of met oxidized α-crystallin was more efficient with 20 µg of TXNL6 compared to 10 µg TXNL6 showing some concentration dependence for TXNL6-mediated MsrA repair of α-crystallin/sHSP. The TXNL6-mediated MsrA repair of α-crystallin/sHSP was less than 100% as estimated by band intensity. This is to be expected since, at most, 50% of mets will be oxidized to form the S-form of PMSO that MsrA specifically recognizes, and the reaction is unlikely to be 100% efficient. Collectively, these studies show that TXNL6 can act as an MsrA reducing system for repair of cyt c and α-crystallin/sHSP that is dependent on the level and reduced state of TXNL6.

## Discussion

It is well accepted that increasing ROS levels during the aging process can cause oxidative damage to cellular proteins whose consequent loss of function contributes to age-onset degenerative disease. To combat damage by ROS, the cell has evolved a diverse range of defenses including specific protein repair systems that restore function to oxidized proteins. Key to the action of these repair proteins is the availability of specific reducing systems required for their catalytic activity. Decreased levels and/or availability of these reducing systems likely limits the activity of oxidative protein repair systems leading to accumulation of oxidized proteins and disease. Identification of those reducing systems and elucidation of the mechanisms that govern their expression and availability under oxidative conditions should provide insight into the regulation and function of repair protein and the etiology of oxidative diseases.

In the present report, we examined the expression of TXNL6 in sub-populations of lens cells, its sub-cellular location in lens cells, its induction under oxidative stress conditions, and its reducing activity for the action of MsrA on two model lens proteins. The results of this study are applicable to understanding the requirements for MsrA function on a wide range of met oxidized target proteins and disease states since methionine is one of the most oxidizable protein amino acids [13] and PMSO accumulation is associated with a broad range of age-related human disorders associated with methionine oxidation including Alzheimer's disease, Parkinson's disease, respiratory distress syndrome, emphysema, and reperfusion injury [1] [2] [3] [4] [5] [7] [14] [15].

Age-related cataract of the eye lens is an excellent model for examining the role of protein oxidation in disease, and the aging eye is an ideal model for identification of those reducing and repair systems that defend against oxidative stress-associated diseases. The lens is a simple structure consisting of only two cell types, it is directly exposed to environmental UV-light, smoke, and other oxidizing agents. It lacks a blood supply and as an encapsulated organ is isolated from the immune system and other systemic influences. Importantly, the cells of the lens and the proteins they contain are not turned over or renewed and therefore must remain un-oxidized for the entire lifetime of an individual for the lens to maintain its transparent function.

One key characteristic of cataract is increased PMSO accumulation which can be repaired by MsrA. MsrA is critical for eye lens function since it is required for oxidative stress resistance in the lens [20]–[21] and prevention of cataract formation [26]. MsrA can repair met oxidized lens proteins and is essential for lens function [20]–[21] [26]–[27]. Deletion of MsrA causes cataract in oxidative stress-treated mice demonstrating its importance in lens defense against oxidative stress [26]. MsrA has demonstrated ability to protect lens cells [20]–[21] and has been shown to repair lens proteins critical for lens function [26]–[27]. Despite the importance of MsrA for lens function, its required reducing system in the lens is not known. Bacterial and yeast MsrA can use bacterial and yeast thioredoxin as a reducing system [61], while other studies suggested that mammalian MsrA may employ reducing systems in addition to or other than Trx [37].

In the present report we provide evidence that in addition to Trx 1 and Trx 2, TXNL6 could be a robust reducing system for MsrA-mediated repair of met sulfoxide in the critical lens proteins alpha-crystallin/sHSP and cyt c, that TXNL6 is expressed at high levels in lens epithelial cells and fiber cells where it co-localizes with MsrA, and importantly, that TXNL6 is induced under the same oxidative conditions that result in oxidation of lens proteins, apoptosis of lens cells, and other events leading to cataract formation. We chose to examine TXNL6 as a potential reducing system for the action of MsrA based on recent studies showing its importance in maintaining the viability of retinal cells in mice [38]–[39]. TXNL6 (RdCVF in mice) is expressed as two RNA splicing variants called RdCVFL (long form) and RdCVF (Short form) [38]. The short form of RdCVF lacks the thioredoxin fold and acts as a trophic factor for the growth of cone photoreceptor cells in the mouse retina [38]. Importantly, disruption of the RdCVF gene leads to photoreceptor dysfunction and increased susceptibility to oxidative stress [39], suggesting a role for RdCVFL in protection against oxidative stress damage. This long form of RdCVF contains both the CXXC domain and the thioredoxin fold making it a likely candidate to act as potential reducing system for the action of MsrA repair activity in the eye lens and other tissues. Only the long form of TXNL6 (RdCVFL in mice) was examined in the present study, since the short form lacks the thioredoxin fold believed to be necessary for oxidoreductase activity [39].

In the present study human TXNL6 was expressed in all tissues examined including lens, retina, stomach, kidney, heart, colon, and spleen suggesting that it is ubiquitously expressed in human tissue. In the eye lens, we detected higher TXNL6 levels in the fiber cells relative to the epithelium (Figure 1 and 2). Since the lens fiber cells have to last throughout the lifetime of the individual and contain some of the oldest cells in the body, the presence of TXNL6 in the lens fibers supports its potential role in MsrA repair of met oxidized proteins since MsrA is also located in the lens fibers [20] and oxidation of fiber cell protein is believed to be a crucial initiator of cataract formation [16]. The low levels of TXNL6 in the epithelium could indicate it is expressed only under oxidative stress conditions in this lens sublocation. There is some evidence that lens cells grown in culture at 20% oxygen are indeed stressed relative to the lower oxygen conditions of the lens *in vivo*. Expression of TXNL6 in the lens epithelium suggests that TXNL6 might act to facilitate MsrA action in the mitochondria as well as the cytoplasm of lens epithelial cells where MsrA has previously been localized [26]. Consistently, sub-cellular localization of TXNL6 using western blot analysis and immunofluoresence staining revealed that TXNL6 was located in both the cytoplasm and the mitochondria of lens epithelial cells (Figure 3 and 4). These results suggest multiple roles for TXNL6 in the eye lens including epithelial metabolism and maintenance of fiber cells.

MsrA has been shown to be critical for lens mitochondrial function [21] and TXNL6 may serve as a reducing system for MsrA in the mitochondria of lens epithelial cells based in its co-localization with MsrA. Strikingly, we also found that TXNL6 is induced by exposure of lens cells to H_2_O_2_-induced oxidative stress (Figure 5) with both the mRNA and protein levels of TXNL6 significantly increasing over 24 h following exposure to 200 µM H_2_O_2_ (Figure 5). Previous studies have shown that TXNL6 expression is associated with upregulation of NFκB activity in response to photooxidation in cone photoreceptor cells [62], suggesting that induction of TXNL6 may be a universal response to defend multiple tissues against acute oxidative stress damage through its activation of MsrA and potentially other defensive and repair proteins.

To examine whether TXNL6 can act as a reducing system for MsrA, we evaluated its ability to act as a reducing system for the specific MsrA-repair of met 80 sulfoxide of the lens epithelial protein cyt c and the specific MsrA-repair of oxidized met 138 (αA-crystallin subunit) and met 68 (αB-crystallin subunit) in the predominately lens fiber protein α-crystallin/sHSP. Cyt c is critical for mitochondrial respiration and oxidation at met 80 results in loss of its electron transport function, increased peroxidase activity [26] [58] and cyt c mediated apoptosis [59]. Previous studies showed that MsrA could repair cyt c oxidized at met 80 using DTT as a reducing system [26]. α-crystallin/sHSP is both a refractive and chaperone protein in the lens required for the maintenance of lens transparency [30]. Previous studies showed that MsrA could repair α-crystallin/sHSP oxidized at mets 138 and 68 using DTT as a reducing system [27]. TXNL6 was able to substitute for DTT in the MsrA repair of met 80 of cyt c and mets 138, 68 of α-crystallin/sHSP (Figures 7 and 8) *in vitro*. Interestingly, repair of cyt c by MsrA using TXNL6 as a reducing agent also decreased cyt c peroxidase activity (Figure 6). In addition, similar to the action of Trx 1 and Trx 2, the ability of TXNL6 to act as a reducing system for MsrA relied on the availability of a reducing enzyme, in this case TxrR. These results provide evidence that in lenses exposed to oxidative stress, increased levels of TXNL6 act to serve as an MsrA reducing system for repair of cyt c and α-crystallin/sHSP in the lens, the maintenance of mitochondrial electron transport, the prevention of cyt c-mediated apoptosis and the maintenance of the chaperone activity of α-crystallin/sHSP required for the prevention of cataract formation. Since TXNL6, Trx 1 and Trx 2 are present in the lens and as shown here act on MsrA, it is likely that they have different functions in the lens. Some of these may be spatially restrictive while other functions may depend on growth and environmental conditions. The induction of TXNL6 demonstrated in the present report (Figure 5) suggests that TXNL6 is the major reducing system for MsrA under oxidative stress conditions and that Trx 2 in the mitochondria and Trx 1 may be more important for MsrA action under non-stressed conditions.

In addition to acting on MsrA it is likely that TXNL6 also acts on other Trx requiring enzymes including peroxiredoxins [63]. TXNL6 is one of a family of thioredoxin-like proteins. Of these, TXNL1, TXNL5 and TXNL6 contain the thioredoxin CXXC domain and therefore could also act on MsrA and as reducing systems for other Trx dependent enzymes. The TXNL2 and TXNL4 members of this family are mono-thiols potentially harboring some regulatory function yet to be elucidated. Other studies [64] have shown that TXNL2 in addition to TXNL6 is present in the lens and we have detected expression of TXNL1 in the lens (unpublished). Future work is needed to examine the effectiveness of the TXNL6 reducing system for MsrA activation relative to other members of the TXNL family, Trx 1 and/or Trx 2, which will likely depend on the cell type, sub-cellular expression pattern, differentiation state, oxidation exposure and specific MsrA target protein examined. Since, Trx 1, Trx 2 and TXNL6 depend on TxrR and NADPH for their activities [63] [65], understanding the potential regulation and expression of TxrR in lens cells upon aging and oxidative conditions is also likely to provide important clues towards understanding the actions of TXNL6 and MsrA in the lens and other tissues. In the lens NADPH is predominantly made in the lens epithelium and possibly the cortical fibers which both contain energy producing mitochondria. Thus, NADPH would likely need transport or diffusion to the inner fibers suggesting that analysis of NADPH levels would also provide insight into the role of TXNL6 and other NADPH-dependent reducing systems on the activities of MsrA.

Regardless of the outcomes of these future studies, the data established in the present report show that TXNL6 is ubiquitously expressed in eye lens and other human tissues. Strikingly, TXNL6 is induced upon oxidative stress conditions which suggests a specific role for TXNL6 in defense of cells against oxidative stress. We also demonstrate that TXNL6 is a novel reducing system for MsrA repair of cellular proteins whose lost functions upon methionine oxidation contribute to eye lens cataract formation. These results provide a greater understanding of the requirements of MsrA for many functions and disease states and likely extend a role for TXNL6 in the function of other cellular protective and repair systems. Finally, recent evidence suggests that the levels of reducing systems are the limiting factors in the function of MsrA in oxidative defense [66]. The results presented in this report demonstrating oxidative induction of TXNL6 suggest it plays a key role in MsrA function under oxidative stress conditions and that control of its induction may be a limiting factor in the ability of MsrA to defend against oxidative stress and disease.
